# Editorial

**Published:** 1882-12

**Authors:** 


### EDITORIAL.

VALEDICTORY.—It is with great reluctance that the Founder and Editor of the INDEPENDENT PRACTITIONER has to retire from editorial work. Other pressing business demands all his time and personal attention ; therefore he gives up the work in which he has labored during the past three years, on many occasions unsatisfactorily to himself and doubtless to his indulgent readers.

Thanking his subscribers for their past forbearance he now with pleasure introduces his successors, who are earnest, eminent and competent. With their ability and energy Vol. IV. cannot fail to surpass in merit and attractive features all its predecessors. Leigh H. Hunt, M. D., of New York City, has undertaken full charge of the medical department. Dr. Hunt, although a young man, has accomplished more than many older members of the Profession, who claim to have literary talent. He graduated with distinction at the New York College—taking the degree of B. Sc., carrying off the chief honors and medals of his class. He subsequently took the degree of M. D. at the University of the City of New York, Medical Department, together with the first prize for general proficiency, as well as prizes for Chemistry and Physiology. Four months later he was appointed Instructor in the Pathological Laboratory of that Institution, a position which he still occupies, with credit to himself and satisfaction to the authorities. He has recently translated Charcot's work on old age, which has proved highly acceptable to the profession.

W. C. Barrett, M. D., D. D. S., of Buffalo, N. Y., has accepted entire charge of the dental department. He is well and favorably known to the dental literary world as a sound and independent thinker, a bold writer and an eminent dental practitioner. Those of our readers practicing the specialty of dentistry are assured that his ability and energy will be fully employed in their service. Physicians will also be interested and edified by reading his selections and original productions.

The writer in making his farewell bow offers an apology to all whom he may have offended either by "omission or commission," be he printer's devil, employee, patron, official or *confrère*. He will be glad to give each, together with all of his friends, a cordial greeting, whenever he may meet them. To all those who have contributed to the pages of the Journal or in any other way rendered service, he tenders his sincere thanks for their co-operation and financial encouragement.

Finally—he hopes that all his friends will continue to support the Journal under its new and most favorable auspices. The child which he has nourished during the past few years, is now (owing to the kind patronage afforded by friends and subscribers) passing from infancy into boyhood, and the Editor feels assured that he leaves it in good hands, surrounded with a large circle of friends, who, like himself, will always cherish an affectionate and hopeful interest in the future success of the INDEPENDENT PRACTITIONER.

With a cordial yet lingering adieu to all, he confidently commits the Journal to the good favor of its subscribers and the blessing of Providence. BASIL M. WILKERSON.

MEDICAL EXCHANGES, ARTICLES FOR PUBLICATION, AND BOOKS FOR REVIEW IN THE MEDICAL DEPARTMENT must be addressed to Dr. Leigh H. Hunt, 411 Lexington Ave., New York City. Extra copies of these exchanges will also be acceptable to the publishers, addressed to 42 Duane St., New York.

DENTAL EXCHANGES, ARTICLES FOR PUBLICATION AND BOOKS FOR REVIEW IN THE DENTAL DEPARTMENT must be addressed to Dr. W.C. Barrett, Dental Editor of the INDEPENDENT PRACTITIONER, No. 11 W. Chippewa Street, Buffalo, N. Y. Those who feel disposed to have their copies on file at the publisher's office will please send them to the Columbia Publishing Co., 42 Duane Street, New York.

POPULAR SCIENCE EXCHANGES AND BOOKS. Please send all books and exchanges for this department to the Columbia Publishing Co., 42 Duane St., N. Y.

DISSOLUTION OF THE INDEPENDENT PRACTITIONER CO..—Having sold our interest to the Columbia Publishing Company, we solicit the support of our patrons to the said Company. Their facilities are superior to any publishing house with which we have dealt, and our readers will, therefore, happily escape the occasional short-comings to which we have unintentionally subjected them.

In order to meet the deficiency of the last few issues of THE INDEPENDENT PRACTITIONER, we send to all our paid up Subscribers a "draw back" certificate for Fifty cents, which will be cashed, or received in part payment for renewed subscriptions.

All those in *arrears* will please settle promptly with the Columbia Publishing Co.

In bidding our many friends adieu, we thank them for their courtesy and encouragement, and bespeak the same liberal support to our successors.

THE INDEPENDENT PRACTITIONER CO.

New York, Dec. 18, 1882.

PUBLISHER'S NOTICE.—We have purchased the whole interest of THE INDEPENDENT PRACTITIONER COMPANY, and entrusted the arduous duties of editorship to thoroughly competent hands. The "Collaborators" will be retained as contributors, but they are in no way answerable for the management of the Journal, further than their own communications, which will always appear under their names.

In assuming the responsibility of publishing THE INDEPENDENT PRACTITIONER, we can, with our ample facilities, guarantee our patrons that we will make the journal *first class in every particular*. Promptness in all our business matters is a specialty with us.

With these assurances we solicit from all reputable Physicians and Dentists a liberal subscription list.

THE COLUMBIA PUBLISHING CO.

New York, Dec. 18th, 1882.

